# First confirmation of imported dengue virus serotype 2 complete genome in urine from a Chinese traveler returning from India

**DOI:** 10.1186/1743-422X-11-56

**Published:** 2014-03-25

**Authors:** Xuezheng Ma, Wei Zhen, Pengfei Yang, Xiaohong Sun, Weizhong Nie, Liping Zhang, Huanzhou Xu, Kongxin Hu

**Affiliations:** 1 Chinese Academy of Inspection and Quarantine, Institute of Health Quarantine, No. A3, Gaobeidian Bei Lu, Chaoyang District, Beijing 100123, China; 2 People’s Republic of China Qinhunagdao Entry-Exit Inspection and Quarantine Bureau, No. 51, Haibin Rd, Qinhuangdao, Hebei 130300, China

**Keywords:** DENV-2, Dengue virus serotype 2, Imported disease, Complete genome, Dengue fever, Secondary infection, Travel-associated disease, Urine

## Abstract

Dengue virus (DENV) is a mosquito-borne virus that has four serotypes. Collection of serum from patients is time- and labor- consuming, and presents a high injury risk for infants and children. The genomic and serological diagnosis of imported dengue fever from a urine sample was used as a non-invasive diagnostic method in this study. A serum sample was collected on disease day 5, and a serum and urine sample were collected on disease day 8 and 18. The results of serological tests for DENV IgM revealed that the serum samples were positive for DENV. The results of RT-qPCR assay revealed that the serum sample collected on day 5 was DENV-positive; however, the serum sample collected on day 8 and 18 were negative for DENV. The urine sample collected on day 8 and 18 were DENV-positive. We also sequenced the complete DENV genome (10723 bp) from the urine sample (GenBank KF479233). The results of phylogenetic and epidemiological analysis indicated strong confirmation that the strain was located within the DENV-2 group with a 100% bootstrap value. In this report, we (1) provided the first evidence of a DENV infection that was imported from India to a non-endemic city of China, (2) investigated the DENV genome detection having a longer timeframe for positive detection in urine sample compared to previous studies, (3) provided the sequence results for the complete DENV-2 genome from a concentrated urine sample (4) discussed how virus-typing results could be used to manage the risk of sero-specific and re-infected travel-associated dengue fever.

## Background

Dengue virus (DENV) has four different serotypes and any one of related types will cause dengue infection with the main transmission of mosquitoes [1]. DENV infection with one serotype may drive the development of dengue hemorrhagic fever (DHF) or dengue shock syndrome (DSS) after re-infection with another serotype, which has a high mortality rate (1–2.5%) [2]. To prevent re-infection, it is important to detect the primary DENV serotype infection in travelers returning from highly endemic areas. The laboratory DENV diagnoses include viral isolation, viral antigen detection, antibody detection and genomic test. The combination of serological tests and RT-qPCR is an approach that is often to confirm DENV infection [3]. Laboratory diagnostic detections in health examination for international travelers usually need patients’ serum as detecting samples. However, collection of serum sample is time- and labor-consuming, presents a high injury risk for infants and children, and depends on patients’ compliance. Several studies have reported that DENV genome detection in urine is an alternative non-invasive diagnostic method, and the timeframes for positive detection in urine are longer than they are in serum [4-6]. Considering the severe DENV secondary infection, the DENV long-last timeframe in urine sample provides a monitoring chance rather than the serological test to detect positive DENV infection with the identification of specific serotypes. This case study reported a longer timeframe for positive detection of dengue virus in urine samples than previous studies have reported. In addition to the difficulties of patients’ serum collection, the weakness of serological test is a high risk of the cross-reaction with other flaviviruses such as Yellow Fever Virus (YFV) [7]. The complete DENV genome provides a strong molecular evidence to determine dengue virus and its serotype. Here, this case report first represents the diagnosis and serotyping of a case of imported dengue fever from the urine sample and provide the full genome sequence of this DENV-2 at a point of entry in North China.

## Case presentation

**Figure 1:**
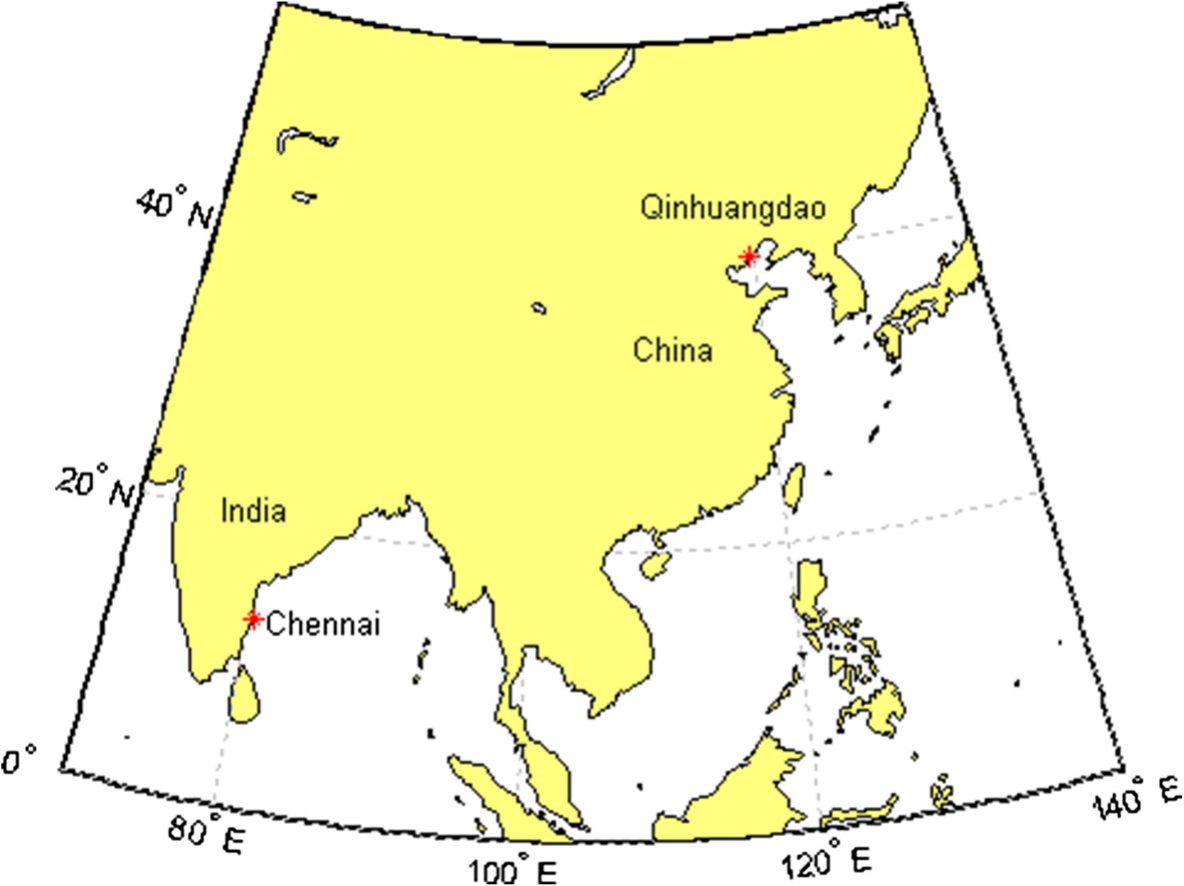
**Location of the case report.** Qinhuangdao: the home city of the patient; Chennai: the travel destination of the patient. Image supplied by MATLAB®.

A 47-year-old businessman who resided in Qinhuangdao, Hebei Province, China, visited India, and then returned home. Domestic DENV infection does not occur in Qinhuangdao, which is located at 39° north latitude (Figure 1). In Asia, the geographical distribution of DENV infection is between 25° north latitude and 25° south latitude, and is concentrated in south central and Southeast Asia (Figure 1) [8].

The patient had no previous medical history of dengue fever or renal disease, and had not received Japanese encephalitis or yellow fever vaccines. He visited Chennai, India, for 5 days, from Dec 18 to Dec 22 2013, and arrived in Qinhuangdao on Dec 23. The patient recalled that he had been bitten by mosquitoes sometime between Dec 19 and Dec 22. If disease day is defined as the time of the fever onset, he developed fever (37.5–39.5°C), arthralgia, retro-orbital pain, waist pain, and abdominal pain on Dec 27 (disease day 0). His clinical examination data were: fever (39.2°C); face exanthema; white blood cell count, 3.8 × 10^9^/L (reference range, 4–10 × 10^9^/L); platelet count, 31 × 10^9^/L (reference range, 100–300 × 10^9^/L); and urine protein +++. He had no previous history of renal disease, blood urea nitrogen, creatinine, β2-microglobulin levels, and urinalysis results were within normal limits from Dec 27, 2013 to Jan 14, 2013. No apparent renal dysfunction was present. By Jan 4, 2013 (day 8), his headache, waist pain, and abdominal pain subsided, and no rashes or hemorrhage were present. There was no bleeding or bruising under the skin, and no pachyemia, or hepatomegaly.

As soon as the results of the clinical and epidemiological investigations indicated the presence of an imported infectious disease, the hospital notified the bureau. Serum and urine samples were collected on Dec 27, 2012 to Jan 14, 2013, stored at -80°C and sent to our laboratory. The RNA samples were extracted from a 5 mL urine sample and were ultracentrifuged at 303800 RCF (BECKMAN COULTER® Optima L-100 XP ultracentrifuge, Fullerton, CA, USA) for 2 hours at 4°C. The total RNA from 140 μl serum and urine samples were extracted separately using the QIAamp Viral RNA Mini kit (QIAGEN, Valencia, CA, USA). The RNA was eluted in 60 μl elution buffer and stored at -80°C. The singleplex RT-qPCR was performed using an Applied Biosystems® 7500 real-time PCR system (Applied Biosystems® by Life Technologies, Carlsbad, CA, USA) to test the DENV conservative region at 3′UTR. The DENV-specific primers and Taqman probes were designed by previous study [9] (Table 1). The RT-qPCR reagents were carried out by AgPath-ID™ One-Step RT-PCR Kit (Ambion, Applied Biosystems® by Life Technologies, USA). The total 25 μl RT-qPCR mixture included 12.5 μl 2X RT-PCR Buffer, 1 μl 25X RT-PCR Enzyme Mix, 0.5 μl one-step RT-PCR Master Mix (Qiagen), 0.5 μl (20 μM ) of each primer, 0.3 μl (10 μM ) probe, 4.7 μl nuclease-free water and 5 μl extracted RNA. The thermocycling parameters were: reverse transcription (RT) at 45°C for 10 min, RT inactivation at 95°C for 10 min and fluorescence detection for 40 cycles of 95°C for 15 s and annealing at 60°C for 45 s. The RT-qPCR data was analyzed using the SDS software provided by Applied Biosystems®. Amplification curves were evaluated by the threshold line placed over the background signal, and intersecting the initial exponential phase of the curve. Amplification of DENV was observed at a quantification cycle (Cq) value of 28. The serological tests for DENV IgM provided in a commercial ELISA kit (Panbio® Dengue IgM Capture ELISA, Sinnamon Park, Australia) were tested from disease day 0 to 18. The NS1 antigen colloidal gold test was carried out using the CIK DENV OnSite Rapid Test® (CTK Biotech, Inc, San Diego, CA, USA). This test was used first as the most rapid diagnostic tool to identify dengue virus. However, the immunochromatographic card test usually has a low specificity and sensitivity compared with molecular methods and ELISAs. The results were consistent with the conclusions of previous studies that demonstrated the compared clinical diagnostic methods [10-12]. The results of the assays were presented in Table 2. The Qinhuangdao Entry-Exit Inspection and Quarantine Bureau advised the patient about future travel in dengue high risk regions before his discharge from the hospital on Jan 15, 2013.

**Table 1:** Nucleotide sequence of primers and probe used in the RT-qPCR assay*

	**Sequence**	**Nucleotide position**
Forward primer	5′-FAM-GARAGACCAGAGATCCTGCTGTCT-3′	10635–10658
Reverse primer	5′-FAM-ACCATTCCATTTTCTGGCGTT-3′	10708–10682
TaqMan MGB probe	5′-FAM-AGCATCATTCCAGGCAC-3′	10663–10679

*: [9].

**Table 2:** Results of real time RT-PCR and serum IgM tests

**Date**	**Disease day**	**Real time RT-PCR**		**Serum antibodies (IgM)**	**NS1 antigen colloidal gold test**
		**Urine**	**Serum**		
2012-12-27	0	NT	+	+	NT
2013-1-1	5	NT	+	+	NT
2013-1-4	8	+	-	-	-
2013-1-14	18	+	-	-	-

NT: No Test; + Positive; - Negative.

The viral RNA was reverse transcribed using the reverse primer with AMV reverse transcriptase (Promega Corporation, Madison, WI, USA). The reverse transcription reaction was carried out at 42°C for 1 h. Further conventional PCR amplification covering the full genome with 16 sets of DENV specific primers succeeded in cDNA template. Amplified products were detected using agarose gel electrophoresis and sequence identification. The sequencing was performed using an ABI PRISM 3730 DNA Sequencer. Overlapping sequencing fragments were assembled to generate a contiguous full-genome sequence (10723 bp) using Lasergene (DNASTAR, Madison, WI, USA). The complete DENV genome was submitted to GenBank (accession number KF479233).

The assembled sequence isolated from the patient’s urine was aligned with NCBI BLASTn default parameter against the non-redundant nucleotide database (nr/nt). An E-value of 0 was used as the cutoff. The cutoff of the query coverage and the identity were >99% and >95% above, respectively. The result indicated that all query hits were dengue virus type 2, but the hits were variant strains from different South Asian countries.

**Figure 2:**
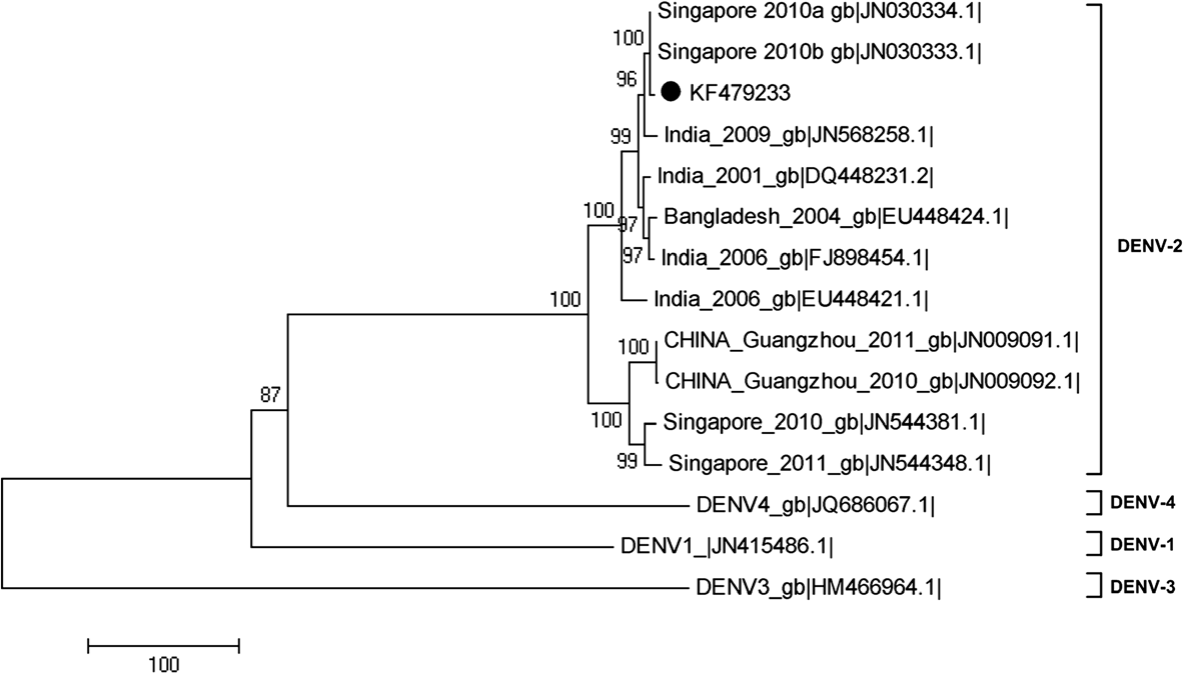
**Neighbor-joining phylogenetic tree based on dengue virus: DENV-1, DENV-2, DENV-3, and DENV-4.** Each taxon indicates a single E gene coding sequence and is labeled with the origin, collection year, and GenBank accession numbers. Boldface taxon label represents the E gene coding sequence from DENV complete genome isolation from a urine sample with the GenBank accession number KF479233. Numbers on each branch represent the bootstrap values.

A phylogenetic analysis of the DENV envelope gene (E gene) coding sequence (1485 bp) was performed by comparing the KF479233 strain with 14 other E gene sequences isolated from four South Asian countries that were obtained from the NCBI GenBank database. The coding sequences were analyzed to make a neighbor-joining tree with 1000 bootstrap replications, which was created using MEGA5 (Tempe, AZ USA) software [13]. After the phylogenetic analysis, DENV-2 strains were separated from the DENV-1, DENV-3, and DENV-4 clades (Figure 2). The KF479233 strain was strongly located within the DENV-2 group, as indicated a high bootstrap value (100%).

## Conclusions

Besides the early clinical diagnosis at the onset of symptoms, our results indicated that urine sample analysis was a noninvasive diagnostic approach, and the timeframes for positive detection in urine were longer than that in serum [6]. Serum samples can have a high detection rate (>50%) in the first week after the onset of infection. However, the last positive detection of virus in serum samples was no later than disease day 11. Urine sample detection rates can be >50% between disease day 6 to 16, with the last positive detection of virus on day 16 [6]. In this case, our data were consistent with previous studies that reported a longer timeframe of positive detection (Table 2). The full DENV-2 genome was sequenced from a urine sample on disease day 18, which was the longest timeframe reported for DENV positive detections. Based on the results of previous studies, the application of DENV detection from urine samples has an important future role for a fast, accurate and noninvasive diagnosis [4,6,14].

Data on the geographical and phylogenetic relatedness of DENV strains provide further evidence on between-country transmission of imported strains. Phylogenetic analysis provided strong evidence to confirm that the sample was associated with the DENV type 2 India group. The most similar strain to the KF479233 strain was isolated in Singapore (bootstrap values, 100%), and indicated that imported from India [15]. The second closest strain to the KF479233 strain was isolated in India in 2009 (JN568258.1); it has been confirmed as an imported case in Australian residents returning from India [16]. DENV-2 was emerged as the prominent serotype outbreaks in Chennai, Tami Nadu, from the 1970s to the 2000s (Figure 1) [17]. The high endemic DENV outbreaks were mostly observed during the rainy season (Oct to Dec) in Chennai, which coincided with the time of travel for the patient in this report [18]. The phylogenetic and epidemiological analysis confirmed that the KF479233 serotype was the DENV-2 imported from India.

DENV infection can develope into two types of clinical illness, dengue fever and more severe DHF or DSS. The first single serotype infection will result in a lifelong immunity to the infecting serotype. During a heterotypic DENV secondary infection, the cross-reactivity will enhance DENV replication and may cause severe DHF or DSS. Although secondary infection by any of the DENV serotypes may cause a severe symptom, not all infections present the same risk for severe development. Early studies of DHF in Thailand suggested that secondary DENV-2 infection was 5–7 times more frequent compared with secondary DENV-1, DENV-3 or DENV-4 infection [19]. As the co-circulation of multiple DENV serotypes in endemic dengue areas, in cases of international travelers, there are more chances of exposure to a cross-reacting transmitted DENV infection. The identification of DENV serotypes present in a primary infection will provide a clear description of the clinical diagnosis for clinicians, and play an important role as an evidence supports for understanding of the mechanism of DHF/DSS and the research of cross-reactivity of different serotype antibodies in a secondary infection. As the recent research and studies reported, there is still no vaccine against DENV and no medications to treat a dengue infection. The prevention will be the most important step, and it means avoiding mosquito bites if re-traveling to an dengue endemic area. The dengue serotype-specific risk needs to monitor against re-infection on the pre-traveling advice for international travelers who will stay in dengue high risk regions in the future traveling.

In this report, we (1) provided the first evidence of a DENV infection that was imported from India to a non-endemic city of China, (2) investigated the DENV genome detection having a longer timeframe for positive detection in urine sample compared with previous studies, (3) provided the sequence results for the complete DENV-2 genome from a concentrated urine sample, and identified DENV-2 by comparing it to isolates from nearby geographic areas, (4) discussed medical advice on the risk of sero-specific and re-infected travel-associated dengue fever based on the virus-typing results.

## Consent

Written informed consent was obtained from the patient for publication of this Case Report and any accompanying images. A copy of the written consent is available for review by the Editor-in-Chief of this journal.

## Competing interests

The authors declare that they have no competing interests.

## Authors’ contributions

XM carried out the sequence analysis and drafted the manuscript. ZW carried out the virus sequencing and assembling. PY, XS, LZ and HX participated in the RNA extraction, RT-qPCR and serological tests. WN provided clinical samples. KH designed the study, edited the manuscript and supervised the experiment. All authors read and approved the final manuscript.
